# The Relationship Between Parent and Child Anxiety: Testing the Intergenerational Model of Anxiety in Children Born with a Cleft

**DOI:** 10.1177/10556656261422883

**Published:** 2026-03-23

**Authors:** Roberta Button, Fiona McFarlane, Alice Farrington, Matthew Hotton

**Affiliations:** 1 The Oxford Institute of Clinical Psychology Training and Research, 6396 University of Oxford, Headington, Oxford, UK; 2 CAMHS Anxiety and Depression Team, Children, Young People and Families, 1900 Berkshire Healthcare NHS Foundation Trust, Bracknell, UK

**Keywords:** children, parents, cleft, anxiety, parenting, appraisals

## Abstract

**Objective:**

To compare overprotective parenting and child and parent anxiety between children born with and without a cleft lip and/ or palate (CL/P). To understand correlates of anxiety for children born with a CL/P and their parents.

**Design, Participants, Setting:**

Parents of 8- to 12-year-old children with a CL/P (*n* = 63, mean age = 10.5 years, male = 68.3%), or without a CL/P (control group; *n* = 66, mean age = 9.9 years, male = 47%) were recruited from the UK community (2022-2024). Most respondents were mothers (92.2%).

**Outcome measures:**

Parents completed an online survey assessing overprotective parenting, child anxiety, parent anxiety, and (in the CL/P group) parental appraisals.

**Results:**

Parent-reported child anxiety was higher in the CL/P group than the control group (η_p_^2^ = 0.04, *P* = .03), while parent anxiety (η_p_^2^ = 0.01, *P* = .26) and overprotective parenting (η_p_^2^ = 0.001, *P* = .73) did not differ significantly between groups. Within the CL/P group, parent-reported child anxiety was associated (*P* < .05) with parental anxiety (*r_s_* = .27), overprotective parenting (*r_s_* *=* .38), and certain parental appraisals (child's self-confidence; *r_s_* *=* .45, and manageability of the CL/P; *r_s_* *=* −.25). Parent anxiety was associated with overprotective parenting (*r_s_* = .40), and self-blame appraisals (*r_s_* *=* .37).

**Conclusions:**

Children born with a CL/P may be at increased risk of anxiety by parent-report, while parental anxiety and overprotection appear comparable to controls. Parental appraisals may be promising targets for intervention. Future longitudinal research is required to draw causal inferences.

## Introduction

Cleft lip and/ or palate (CL/P) is the most common congenital anomaly of craniofacial structure, affecting 1 in 1000-1500 live births globally, and is more common in males than females,^1,2^ Children born with a CL/P usually undergo surgery within the first year of life for their lip and/ or palate, with later surgeries sometimes required for speech, aesthetic and other functional purposes.^3^ Children and young people (CYP) born with a CL/P and their families can face challenges such as feeding difficulties, ear and hearing issues, speech difficulties, issues related to living with a visible difference, and psychosocial distress.^4^ For these reasons, National Health Service (NHS) guidelines state that CL/P should be treated by a multi-disciplinary team (MDT) made up of specialist nurses, speech and language therapists, dentists, audiologists, surgeons, and clinical psychologists.^5^

The literature regarding psychosocial outcomes for CYP born with a CL/P is generally mixed. Some research indicates that being born with a CL/P increases the risk of difficulties, including low self-esteem, anxiety, and depression.^6,8^ Research has specifically suggested that children born with a CL/P are at an increased risk of separation anxiety disorder in childhood, social anxiety disorder in adolescence, and social interaction difficulties.^7,9,10^ However, there is also evidence to the contrary, that the impact of being born with a CL/P on psychological well-being is generally low, and that psychological outcomes for those born with a CL/P are comparable to those of the general population.^6,10,11^ The range of findings in the literature may be due to differences in the local context of samples, and the timeframe when studies were completed, as well as the complex interaction between CL/P-related factors, such as difficulties with speech and feeding, poor satisfaction with speech and appearance,^7,9^ and non-CL/P-related factors, such as lower socioeconomic status, maternal stress, and a comorbid condition.^9,12–14^ The role of gender has also been investigated, with mixed findings depending on the outcome being measured. Boys with a CL/P are at increased risk of behavioral difficulties compared to girls, and girls report higher levels of emotional difficulties compared to boys, a finding that is similar to the general population.^15,16^

The outcomes with regard to parental mental health are also highly variable, with some research finding that parents of children born with a CL/P are at an increased risk of depression, anxiety, post-traumatic stress, and reduced quality of life compared to parents of children without a CL/P, whereas other research has found no difference in psychological outcomes between these groups.^17,18^

There is evidence to suggest that parental anxiety in this group is driven by CL/P-related concerns, with feeding and speech concerns being highest among parents of children born with a CP, and appearance concerns being highest among parents of children born with a CL.^19,20^ Qualitative research highlights that parents feel guilty and conflicted about the need for intervention while wanting to reduce distress for their children.^21^ Parents anticipate that their child will be stigmatized throughout life for their perceived difference and, as a result, find it hard to balance the desire to protect their child from stigma with the need to encourage independence.^21^

The stress-coping model can help explain why having a child born with a CL/P is associated with anxiety in some, but not all, parents.^22^ This model suggests that parental anxiety would be strongest when threat appraisals outweigh coping appraisals, and is supported by research suggesting that parental appraisals of their child's CL/P predict variance in parental well-being, independently of demographic and social support variables.^23^

### The Intergenerational Model of Anxiety

The presence of heightened levels of anxiety in some children born with a CL/P and their parents is an important area of study, as research and theory suggest a link between parent and child anxiety.^24,25^ The intergenerational model of anxiety (IMA) suggests that parent anxiety is related to appraisals that overestimate threat and underestimate coping and that these, in turn, lead to fear-enhancing and lack of autonomy-granting behaviors, which include overprotective parenting practices. These parenting practices inadvertently promote anxious cognitive styles in children, which lead to feelings of anxiety.^24^

### The Intergenerational Model of Anxiety and Cleft Lip and/ or Palate

The IMA has not been evaluated in children born with a CL/P specifically; however, longitudinal research has implicated negative parental appraisals as a causal contributing factor to poorer parental quality of life.^18^ Research also suggests that parent-child interactions in the context of child chronic illness are characterized by higher levels of overprotection.^26,27^ Qualitative data highlights that parents struggle to promote independence due to concerns that their child will be stigmatized and are less able to cope with life transitions.^21^

### Research Aims and Hypotheses

The current study aims to examine the role of overprotective parenting and parental appraisals, and the relationship between parent and parent-reported child anxiety in children born with a CL/P. This project is in-line with recommended research priorities and future directions for clinical psychology research in CL/P care, which have been co-developed with individuals born with a CL/P, their family members, and clinicians. Namely, these priorities highlight the importance of understanding the impact of a CL/P on individual and family well-being (future direction 1), and understanding factors that predict psychosocial adjustment in this group (future direction 2).^28^

*Research Question 1.* Comparing overprotective parenting between the CL/P and control group.

The primary research question asks whether parents of children born with a CL/P (“the CL/P group”) report more overprotective parenting compared to parents of children without a CL/P (“the control group”).

*Hypothesis 1.* It was hypothesized that participants in the CL/P group would report higher levels of overprotective parenting compared to those in the control group. Further correlational analyses explored what factors were associated with overprotective parenting.

*Research Question 2*. Comparing parent-reported child anxiety (2a) and parent anxiety (2b) between the CL/P and control group.

The second research question asks whether rates of parent-reported anxiety are higher among children in the CL/P group (question 2a) and their parents (question 2b) compared to the control group.

*Hypothesis 2.* It was hypothesized that participants in CL/P group would report higher levels of parent-reported child anxiety and parent anxiety than those in the control group.

*Research Question 3*. Exploring relationships with parent-reported child anxiety and parent anxiety in the CL/P group.

Research Question 3 asks what factors are associated with parent and child anxiety in the CL/P group. As this question is exploratory in nature, no specific hypotheses were made.

## Methods

### Design

This study used a cross-sectional design, following the STROBE (Strengthening the Reporting of Observational Studies in Epidemiology) guidelines.^29^

### Ethics

Ethical approval was obtained from an institutional research ethics committee. Respondents gave informed consent online (via Qualtrics) prior to completing the survey.

### Experts-by-Experience

Three parents of children with a CL/P were recruited through the Cleft Lip and Palate Association (CLAPA) and acted as consultants by providing insights and feedback regarding the study design.

### Participants

Participant recruitment was carried out in the United Kingdom between December 2022 and August 2024. Participants in the control group were recruited through the professional social media platforms of the study team, targeted outreach within UK-based online parenting groups, and recruitment facilitated by schools. For the CL/P group, participants were recruited through the social media channels of CLAPA and targeted outreach within UK-based online CL/P parenting groups.

Participants were eligible for inclusion if they were a parent of a child aged between 8 and 12 years old, could speak English, and consent to the study. Participants were excluded if their child had a neurodevelopmental diagnosis, learning disability, genetic syndrome, or a chronic physical health condition requiring specialist review and/ or treatment. For the CL/P group only, parents of children with a submucous CL/P and/ or a CL/P associated with a syndrome were excluded. A power calculation using G-power indicated that a total sample size between 102 (for a power of 0.8) and 176 (for a power of 0.95) was required to detect a medium effect size of *d* = 0.5 for Research Question 1. A final sample size of 129 was achieved, with 63 participants in the CL/P group and 66 in the control group.

### Measures

*Parent-reported child anxiety*: *The Revised Children's Anxiety and Depression Scale-Parent Version (RCADS-P)* is a 47-item parent-report questionnaire measuring anxiety and depression in children and young people between the ages of 8 and 18 years old.^30^ Parents rate each item on a 4-point Likert scale (0 = never; 1 = sometimes; 2 = often; 3 = always) according to how true each statement is for their child. The scale yields an age-corrected T-score for common childhood anxiety disorders (separation anxiety, generalized anxiety, panic disorder, social phobia, and obsessive-compulsive disorder), as well as depression and a total anxiety scale. For the purposes of this study, the full RCADS-P was administered, but only the anxiety subscales and total anxiety scores were calculated and reported on, as these were the domains of interest for the study. In previous research, the RCADS-P has displayed good convergent validity in non-mental health settings.^31^ For the current sample, the RCADS-P total anxiety showed excellent internal consistency for both the control (α = 0.95) and CL/P groups (α = 0.96).

*Parent anxiety: The Generalized Anxiety Disorder (GAD-7) questionnaire* consists of 7 self-report items asking about anxiety symptoms over the past 2 weeks. Respondents rate the frequency of various symptoms from “not at all” to “nearly every day.”^32^ The GAD-7 has shown good reliability and good construct, criterion, and procedural validity.^32^ For the current sample, the GAD-7 showed excellent internal consistency for both the control (α = .92) and CL/P groups (α = .90).

*Parent overprotection: The Parental Overprotection Measure*^33^ (OPM) is a 19-item self-report questionnaire containing situation-specific questions, designed to assess the extent to which parents engage in behaviors that restrict a child's independence. Parents rate the extent to which the statement represents their typical behavior on a 5-point Likert scale (0 = not at all; 4 = very much). The OPM has shown very good internal consistency and predictive validity in a sample of 7- to 12-year-olds.^33^ In the current research, the OPM showed very good internal consistency (α = .89 for both groups)

*Parent appraisals of CL/P:* The *Parental Appraisals of Cleft-Questionnaire (PAC-Q)* is a 30-item parent-report of appraisals of their child's CL/P.^23^ Parents rate the extent to which they agree with statements on a scale of 0-100 (0 = “I do not believe this statement at all”). The items map on to 5 subscales, listed here with Cronbach's alpha for the current sample alongside the interpretation; (1) Child's Self-Confidence (α = .77; a high score indicates more negative parental beliefs regarding their child's self-confidence), (2) Self-blame (α = .87; a high score indicates stronger feelings of blame), (3) Growth through Challenge (α = .89; a high score indicates stronger beliefs of a positive outcome), (4) Perceived Manageability (α = .45; a high score indicates stronger beliefs that their child will be less affected by the CL/P), (5) Impact of Treatment (α = .71; a high score is indicative of stronger negative beliefs that treatment will be experienced as distressing or difficult). In other research, the PAC-Q has shown good test-retest reliability and good convergent and divergent validity.^23^

*Perceived support from child's CL/P team:* Participants in the CL/P group were asked to rate the extent to which they feel supported by their child's CL/P team (“how supported do you feel by your child's Cleft team?”) on a scale of 0 to 100 (100 = very supported). This idiosyncratic measure was included to assess the extent of the association between subjective feelings of support and other variables.

### Procedures

An online study format was used, with Qualtrics hosting the entire study, including the information sheet and consent form. The study advert and link were disseminated via the professional social media channels of the study team, posted in online parenting groups, shared through school networks, and, for the CL/P group, was posted on the social media channels of CLAPA.

### Data Analysis

Data were analyzed using Statistical Package for the Social Sciences (SPSS^®^ 29.0; IBM Corporation, Armonk, NY, United States). Group comparisons of demographic data were conducted using Chi-squared for categorical data and the Mann-Whitney *U* test for child age, as this was non-normally distributed. So as not to violate the chi-squared assumption regarding minimum expected observations, ethnicity was coded as “white” versus “other,” and family structure was coded as “two-parent family” versus “other.” It was not possible to conduct comparisons for “relationship to child” and “parent gender” variables, as these would have violated the minimum expected observation assumption. Tests for underlying assumptions were carried out, and there was found to be some degree of non-normality in some of the continuous measures (see Supplementary materials), but it was deemed that parametric tests for group comparisons to be sufficiently robust, due to the Central Limit Theorem.^34^

Analysis of covariance (ANCOVA) tests were used to assess the between-group differences hypothesized in Research Questions 1, 2a, and 2b. For all analyses, group (CLP vs. control) was entered as a fixed factor and gender as a covariate to account for group differences in gender. Total OPM scores (for Research Question 1), RCADS-P scores (for Research Question 2a), and GAD-7 scores (for Research Question 2b) were added as dependent variables. Additionally, to test for group differences between the proportion of participants with clinically significant anxiety symptoms, scores on the RCADS-P and GAD-7 were coded as either above or below their recommended clinical cut-off of 70 and 10, respectively. A chi-squared analysis was conducted to compare the proportion of participants who scored above the clinical cut-off for these measures between groups.

The validity of the IMA was assessed in Research Question 1, whereby Spearman's rho correlations were conducted across both groups to assess the relationship between OPM and RCADS-P scores, and OPM total scores and GAD-7 scores. For the CL/P group only, a Spearman's rho correlation was conducted between OPM total scores and each of the PAC-Q subscales.

For Research Question 3, data from the CL/P group only were included in Spearman's rho correlations between parental OPM, RCADS-P, GAD-7, and PAC-Q scores, and the “feelings of support” measure. Finally, a *t*-test was used to analyze gender differences in anxiety within the CL/P group, where gender was entered as the independent variable and RCADS-P *t*-scores were entered as the dependent variable.

Effect sizes are interpreted according to Cohen's guidelines and are reported as follows^35^; Cohen's *d* (*d*) for *t*-tests (.20 = small effect, .50 = medium effect, .80 = large effect), partial Eta-squared (η_p_^2^) for ANCOVAs (.01 = small effect, .06 = medium effect,.14 = large effect), Phi φ for chi-squared tests (.10 = small effect, .30 = medium effect, .50 = large effect), and Spearman's rho (*r_s_*) for correlations (.10 = small effect, .40 = medium effect, .70 = large effect).

## Results

### Participant Characteristics

Participant characteristics are summarized in Table 1. There were no significant differences between the CL/P and control groups in terms of child's age (*t*(127) = −0.48, *P* = .63, *d* = −.08), child ethnicity, χ^2^(4, 129) = 2.4, *P* = .66, φ = 0.14, presence of siblings, χ^2^(1, 129) = 0.13, *P* = .72, φ = −0.03, family structure, χ^2^(1, 129) = 0.67, *P* = .41, φ = −0.07, or transition to secondary school in the last 12 months, χ^2^(1, 129) = 0.4, *P* = .53, φ = 0.06. Child gender differed significantly between groups, χ^2^(1, 129) = 5.97, *P* = .02, with a small to medium effect (φ = 0.22) whereby the CL/P group had a higher proportion of boys to girls than the control group. Within the CL/P group, 10% (*n* = 6) had a CP, 21% (*n* = 13) had a CL, 41% (*n* = 26) had a unilateral CLP, and 29% (*n* = 18) had a bilateral CLP. Parental ratings of “support from CL/P team” ranged from 9 to 100 (out of 100, where 100 = very supported), with a mean of 76.74 (*SD* = 22.87).

*Research Question 1.* Comparing overprotective parenting between the CL/P and control group.

**Table 1.** Participant Characteristics.

		Control Mean (*SD*)/*n* (%)	CL/P Mean (*SD*)/*n* (%)	Total Mean (*SD*)/*n* (%)
Child age (years)		9.9 (1.23)	10.05 (1.52)	9.9 (1.38)
Child gender	Girl	35 (53%)	20 (31.7%)	55 (42.6%)
	Boy	31 (47%)	43 (68.3%)	74 (57.4%)
Child ethnicity	White	81.8% (*n* = 54)	88.9% (*n* = 56)	85.3% (*n* = 110)
	Mixed/multiple	7.6% (*n* = 5)	3.2% (*n* = 2)	5.4% (*n* = 7)
	Asian/Asian British	6.1% (*n* = 4)	4.8% (*n* = 3)	5.4% (*n* = 7)
	Black/African/Caribbean/Black British	3% (*n* = 2)	3.2% (*n* = 2)	3.1% (*n* = 4)
	Other	1.5% (*n* = 1)	0% (*n* = 0)	0.8% (*n* = 1)
Secondary school transition in the last 12 months	Yes	9.2% (*n* = 6)	12.7% (*n* = 8)	10.9% (*n* = 14)
	No	90.8% (*n* = 59)	87.3% (*n* = 55)	89.1% (*n* = 114)
Presence of siblings	Yes	86.4% (*n* = 57)	84.1% (*n* = 53)	85.3% (*n* = 110)
	No	13.6% (*n* = 9)	15.9% (*n* = 10	14.7% (*n* = 19)
Relationship to child	Mother	90.9% (*n* = 60)	93.7% (*n* = 59)	92.2% (*n* = 119)
	Father	7.6% (*n* = 5)	6.3% (*n* = 4)	7% (*n* = 9)
	Stepmother	1.5% (*n* = 1)	0% (*n* = 0)	0.8% (*n* = 1)
Parent gender	Female	92.4% (*n* = 61)	93.7% (*n* = 59)	93% (*n* = 120)
	Male	7.6% (*n* = 5)	6.3% (*n* = 4)	7% (*n* = 9)
Family structure	Two-parent family	80.3% (*n* = 53)	85.7% (*n* = 54)	82.9% (*n* = 107)
	Single parent	13.6% (*n* = 9)	9.5% (*n* = 6)	11.6% (*n* = 15)
	Other	6.1% (*n* = 4)	4.8% (*n* = 3)	5.4% (*n* = 7)

There was no significant difference between the CL/P (*M* = 28.68, *SD* = 12.54) and control groups (*M* = 28.35, *SD* = 11.67) on parental overprotection, *F*(1, 126) = 0.12, *P* = .73, (η_p_^2^ = 0.001). The validity of the IMA model was tested for the whole sample, and results, displayed in Table 2, found that parental overprotection was significantly related to parent-reported child anxiety, *r_s_* = .31, *P* ≤ .001 (small effect size), and parental anxiety, *r_s_* = .37, *P* ≤ .001 (small-to-medium effect size). Parent and parent-reported child anxiety were significantly related, *r_s_* = .38, *P* ≤ .001, with a small-to-medium effect size.

*Research Question 2*. Comparing parent-reported child anxiety (2a) and parent anxiety (2b) between the CL/P and control group.

**Table 2.** Correlations Between Components of the IMA for the Whole Sample.

	1.	2.	3.
1. Parental overprotection measure	—	.31**	.37**
2. RCADS-P total anxiety	.31**	—	.38**
3. GAD-7	.37**	.38**	—

**Correlation is statistically significant at the 0.01 level (2-tailed).

Results for Research Question 2 are displayed in Table 3. While within the average clinical range, there was a significant difference between the CL/P group (*M* = 57.38, *SD* = 16.88) and control group (*M* = 52.52, *SD* = 12.10) on parent-reported child anxiety, *F*(1, 126) = 5.11, *P* = .03, with a small-to-medium effect size (η_p_^2^ = 0.04). Clinical levels of anxiety also differed significantly between the groups, χ^2^(1) = 6.48, *P* = .01, whereby 24% of the CL/P group scored above the clinical cut-off for parent-reported child anxiety, compared to 8% of the control group with a small effect size (φ = 0.24).

**Table 3.** Results of Between-Group Comparisons (ANCOVA Analyses) for Research Questions 1 and 2.

	Total *M* (*SD*)	CL/P group *M* (*SD*)	Control group *M* (*SD*)	*F*	*P*	η_p_^2^ (interpretation)
Overprotective parenting measure	28.51 (12.06)	28.68 (12.54)	28.35 (11.67)	0.12	.73	.001 (small)
RCADS-P total anxiety	54.89 (14.78)	57.38 (16.88)	52.52 (12.10)	5.11	.03*	.04 (small)
RCADS-P separation anxiety	54.93 (14.98)	58.06 (17.24)	51.94 (11.84)	7.59	.01*	.06 (small-to-medium)
RCADS-P generalized anxiety	52.64 (11.35)	54.52 (12.68)	50.85 (9.67)	4.02	.05*	.03 (small)
RCADS-P panic	55.88 (17.40)	57.68 (18.72)	54.15 (15.99)	2.17	.14	.02 (small)
RCADS-P social anxiety	55.36 (13.95)	56.79 (15.05)	53.98 (12.78)	2.37	.13	.02 (small)
RCADS-P obsessive-compulsive disorder	49.97 (10.97)	50.97 (11.90)	49.02 (9.99)	1.33	.25	.01 (small)
GAD-7	6.53 (5.18)	6.00 (4.76)	7.01 (5.52)	1.26	.26	.01 (small)

Higher scores indicate greater overprotective parenting and higher anxiety symptoms.

*Clinically significant result.

With regards to specific areas of parent-reported child anxiety, there were significant group differences on RCADS-P separation anxiety *t*-scores, *F*(1, 126) = 7.59, *P* = .01, with a medium effect (η_p_^2^ = 0.06) of the CL/P group scoring significantly higher (M = 58.06, *SD* = 17.24) than the control group (*M* = 51.94, *SD* = 11.84), though both groups scored within the average clinical range. Similarly for the RCADS-P generalized anxiety *t*-scores, both groups scored within the average range, though the CL/P group (*M* = 54.52, *SD* = 12.68) scored significantly higher than the control group (*M* = 50.85, *SD* = 9.67), *F*(1, 126) = 4.02, *P* = 0.5, with a small-to-medium effect size (η_p_^2^ = 0.03). All other group comparisons between RCADS-P subscale scores were non-significant (see Table 3 for full results).

For Research Question 2b, there was no significant difference in parental anxiety between the CL/P (*M* = 5.95, *SD* = 4.76) and control groups (*M* = 7.09, *SD* = 5.52), *F*(1, 126) = 1.26, *P* = .26 (η_p_^2^ = 0.01) Consistent with this, there was also no significant difference between groups on clinically significant symptoms of anxiety, χ^2^(1) = 2.63, *P* = .11 (φ = −0.14). These results demonstrated that 21% of parents in the CLP group reported clinically significant levels of anxiety, compared to 33% of parents in the control group.

*Research Question 3*. Exploring relationships with parent-reported child anxiety and parent anxiety in the CL/P group.

Results of the correlational analyses, displayed in Table 4, found that parent-reported child anxiety was significantly correlated with parental overprotection (*r_s_* = .38, *P* = .002; small-to-medium effect size), parental anxiety (*r_s_* = .27, *P* = .02; small effect size), parental appraisals regarding *child's self-confidence* (PAC-Q subscale 1; *r_s_* = .45, *P* ≤ .001; medium effect size), and *perceived manageability* (PAC-Q subscale 4; *r_s_* = −.25, *P* = .05; small effect size). The direction of the PAC-Q correlations confirms a relationship between more negative parental appraisals regarding child's self-confidence, the manageability of the CL/P, and higher parent-reported child anxiety. Parent-reported child anxiety did not correlate with any other subscales of the PAC-Q.

**Table 4.** Spearman's Rho Correlations (*r_s_*) for Research Question 3.

	1	2	3	4	5	6	7	8	9
1. Parental overprotection measure	—	.38**	.40**	.20	.26*	.09	−.13	.11	−.07
2. RCADS-P total anxiety	.38**	—	.27*	.45**	.13	−.02	−.25*	.07	−.19
3. GAD	.40**	.27*	—	.23	.37**	.04	−.19	.06	.06
4. PAC-Q 1: child's self-confidence	.20	.45**	.23	—	.37**	−.34**	−.35**	.34**	−.24
5. PAC-Q 2: self-blame	.26*	.13	.37**	.37**	—	.10	−.29*	.20	−.14
6. PAC-Q 3: growth through challenge	.09	−.02	.04	−.34**	.10	—	.14	.08	.00
7. PAC-Q 4: perceived manageability	−.13	−.25*	−.19	−.35**	−.29*	.14	—	−.11	.51**
8. PAC-Q 5: impact of treatment	.11	.07	.06	.34**	.20	.08	−.11	—	−.03
9. Perceived support from medical team	−.07	−.19	.06	−.24	−.14	.00	.51**	−.03	—

*Correlation is statistically significant at the .05 level (2-tailed).

**Correlation is statistically significant at the .01 level (2-tailed).

Parent-reported child anxiety was also found to differ significantly between genders, *t*(61) = 1.73, *P* = .04, with a small-to-medium effect (*d* = 0.47) of girls (*M* = 62.7, *SD* = 13.8) of girls scoring significantly higher for anxiety than boys (*M* = 54.9, *SD* = 17.7).

Parental anxiety was significantly positively correlated with parental overprotection (*r_s_* = .40, *P* = .001) with a medium effect size. Parental anxiety was also significantly positively correlated with appraisals regarding *self-blame* (PAC-Q subscale 2; *r_s_* = .37, *P* = .003) with a small-to-medium effect size, suggesting that stronger beliefs regarding self-blame were associated with higher levels of parental anxiety. No other subscales of the PAC-Q were significantly correlated with parental anxiety.

Finally, there were several other significant correlations of note. Parental feelings of support from the CL/P team were significantly positively correlated with parental appraisals regarding *perceived manageability of CL/P* (PAC-Q subscale 4; *r_s_* = .51, *P* ≤ .001; medium effect size), suggesting that stronger feelings of support from the CL/P team are related to positive appraisals about CL/P manageability. Parental appraisals regarding *self-blame* (PAC-Q subscale 2) were significantly correlated with parental overprotection (*r_s_* = .26, *P* = .04; small effect size).

There were also several significant correlations between PAC-Q subscales. *Child's self-confidence* (PAC-Q subscale 1) was significantly correlated to *self-blame* appraisals (PAC-Q subscale 2), *r_s_* = .37, *P* ≤ .003 (small-to-medium effect size), *growth through challenge* (PAC-Q subscale 3), *r_s_* = −.34, *P* ≤ .01 (small-to-medium effect size), *perceived manageability of CL/P* (PAC-Q subscale 4), *r_s_* = −.35, *P* ≤ .01 (small-to-medium effect size), and *impact of treatment* (PAC-Q subscale 5*)*, *r_s_* = .34, *P* < .01 (small-to-medium effect size). The direction of the correlations indicates that more negative beliefs regarding the child's self-confidence were associated with higher levels of parental self-blame, more pessimistic beliefs about treatment outcomes, more negative appraisals of the child and family's ability to cope, and stronger perceptions of the distressing impact of treatment.

Self-blame (PAC-Q subscale 2) was negatively correlated with perceived manageability, *r_s_* = −.29, *P* = .02 (small effect size), suggesting that higher levels of parental self-blame were associated with more negative beliefs regarding the manageability of the CL/P.

## Discussion

This research aimed to understand parental factors in relation to child anxiety in those born with a CL/P. Results found that parents of children with a CL/P reported similar levels of overprotection and anxiety as those in the control group, and that appraisals regarding self-blame were significantly correlated to both overprotection and parental anxiety in parents of children born with CL/P. Children born with a CL/P had higher levels of parent-reported anxiety than children without a CL/P, and this was driven by significantly higher levels of separation anxiety and generalized anxiety in this group. For the CL/P group, parent-reported child anxiety was associated with parental overprotection, parental anxiety, negative parental appraisals regarding child's self-confidence and the perceived manageability of the cleft, and female gender.

The current study investigated the IMA in children born with a CL/P, and results here support the validity of the model when applied to this group of children and families. Results found that parents of children born with a CL/P can promote developmentally appropriate independence to the same level as parents of children born without a CL/P. While this finding was contrary to our hypothesis, it suggests that parents are able to resolve the emotional tensions of protection vs promoting independence.^21^ The finding that overprotection rose in line with parent and parent-reported child anxiety is consistent with the IMA and general population research, and suggests that overprotection is an important parental behavior in understanding anxiety in children with a CL/P and their parents.^33^ In the CL/P group, the relationship between certain parental appraisals and parent anxiety and parent-reported child anxiety is consistent with IMA predictions. Specifically, in the CL/P group, the finding that parental appraisals regarding self-blame were significantly correlated to parental anxiety is consistent with previous research.^18^ The additional finding that self-blame appraisals were correlated with parental overprotection in the CL/P group provides novel information on the mechanisms by which parental anxiety, appraisals, and behaviors may be linked in parents of children born with a CL/P. The IMA would lead to the interpretation that, in parents of children born with a CL/P, parental anxiety and self-blame appraisals are bidirectionally linked, and that self-blame appraisals are one of the factors that contribute to overprotective parenting.^24^ This interpretation is partially supported by the significant correlation between self-blame appraisals in parents of children born with a CL/P, and overprotective parenting, however, more research is required to determine causality.

The finding that parental anxiety was similar between the CL/P and control group was contrary to our hypothesis and some of the literature but, given the similarity between the 2 groups in terms of overprotection, is in-line with what would be predicted by the IMA.^24^ It is possible that the discrepancy between our findings and some of the literature is due to the complexity of adjustment to a CL/P, which is complicated by various dynamic and static risk and protective factors, including social, cultural, physical, and psychological factors.^36^ One possible factor when considering parental outcomes is the age of the child. Qualitative data suggests that the first year of life is experienced as the most distressing for parents,^37,38^ while longitudinal research has found that parental quality of life significantly improves over the course of time.^18^ It is therefore possible that the risk of anxiety fluctuates over the course of parenting a child with a CL/P, and that the 8-12-year-old age bracket represents a period of lower risk for parental anxiety, however, longitudinal research would be required to test this hypothesis.

The context where this research was conducted is also worth considering. In the United Kingdom, children and families are supported by a specialist CL/P team, which includes clinical psychology.^39^ The significant relationship between how supported parents felt by their child's CL/P team and positive appraisals about treatment manageability suggests that CL/P teams provide a sense of manageability and containment for parents, and it is therefore possible that this support protects parents from anxiety.^17,22^ We would therefore recommend caution in generalizing these findings to other contexts or countries whereby cleft care may be different to the United Kingdom.

The finding that children born with a CL/P are at a small but significantly increased risk of parent-reported anxiety, and that nearly a quarter of these children experience clinically significant levels of anxiety is an important addition to the mixed literature on this topic. This finding is consistent with other literature suggesting that most children born with a CL/P have similar psychological outcomes to their peers, but that a minority of children will experience emotional difficulties.^40^ The finding that parent-reported child anxiety significantly correlated with parental anxiety, parental overprotection, and certain negative parental appraisals provides some indication of possible pathways to anxiety in children born with a CL/P.

The significant correlations between PAC-Q subscales suggest that parental appraisals regarding a child's CL/P are embedded within a broader constellation of parental cognitive evaluations related to their child's CL/P. Results suggest that parental appraisals regarding child's self-confidence are particularly interconnected with other appraisals, with more negative perceptions of child's self-confidence related to greater parental self-blame, more pessimistic beliefs about growth through challenge and treatment outcomes, lower perceived manageability of CL/P, and stronger perceptions of treatment-related distress. This clustering of appraisals is consistent with the IMA, which suggests that parental appraisals regarding a child's ability to cope are linked to parental appraisals regarding threat parental perceptions of coping.^24^

### Clinical Implications

Clinical implications will be discussed according to the Pediatric Psychosocial Preventative Health Model (PPPHM), which proposes a hierarchy of needs and associated interventions (universal, targeted, and specialist) within pediatric settings.^41^ Based on the findings of this study, a Universal intervention in the form of clinical and psychoeducation to assess and challenge unhelpful appraisals is recommended. The results indicate that targeting parental appraisals of self-blame, child's self-confidence, and the perceived manageability of the CL/P may be of particular benefit. However, the finding that parental appraisals were interconnected suggests that there may be some benefit to assessing and targeting a wider range of negative parental appraisals. In the United Kingdom, it is possible that such an intervention be provided by routine contact with CL/P clinical nurse specialists.

Where distress is elevated, as was the case for 24% of the children with a CL/P in this study, clinical psychology interventions drawing on techniques from cognitive behavior therapy (CBT) and compassion-focused therapy (CFT) may be helpful. CFT has been found to be effective in reducing self-blame, self-criticism and shame in parents^42,43^ and parent-led CBT, whereby negative appraisals and overprotective parenting practices have been targeted, has been found to be clinically and cost-effective in reducing child anxiety.^44^ However, the evidence for the efficacy of psychological interventions in the field of CL/P is mixed, and further research is therefore required.^45^

### Limitations

The fact that some comorbid conditions were excluded from this project means that these results cannot be generalized to the general population of children born with a CL/P. This is important because it is reported that 40% of children born with a CL/P have at least one other condition, and that comorbidity represents a significant risk factor for psychological difficulties in this group.^13^

The use of parent-report only carries a risk of bias, as parental reports of child anxiety may be contaminated by parental anxiety and appraisals and may therefore not be a valid measure of child anxiety.^46^ However, the RCADS-P has demonstrated similar sensitivity and specificity compared to gold-standard diagnostic interview, and has shown good agreement with the RCADS child version.^47^

Given the known relationship between socio-economic status and psychological outcomes in children with a CL/P and their families, the lack of inclusion of any measure of socio-economic status in this study limits the internal and external validity.^48^ It is recommended that measures of socioeconomic status be included in future research in this area.

The sample had an unrepresentative distribution of cleft types, namely a larger group of CLP and a smaller group of CP compared to the wider cleft population^1^ which may threaten the validity of the findings given that there is some evidence to suggest that parental concerns differ significantly depending on the type of cleft,^19^ and that children born with a CP have fewer adjustment problems than those born with a CLP.^16^

The cross-sectional design used here meant that inferences about causal relationships were limited; thus, the direction of the relationships between parental factors and child anxiety is unclear. In addition to this, the fact that the project was powered only for the primary research question meant that it lacked sufficient power to conduct mediational analyses to understand the direct and indirect pathways to anxiety in children with a CL/P.

### Future Research

It is recommended that future research investigate the interaction of parent and child factors over time using a longitudinal design and mediational analyses to infer causality. Given the limitations regarding the use of parent—as opposed to child-report, it is recommended that future research include CYP self-reported anxiety. Future research should address other factors related to family and child adjustment, particularly the presence of comorbid conditions and the role of cleft type. Furthermore, it is recommended that research focuses on the efficacy of intervention studies targeting the appraisals and parenting behaviors identified as possible contributors to parent and child anxiety. Such studies could look at intervention at various stages of the PPPHM, assessing the impact of Universal psychoeducational approaches on parental appraisals and behaviors, as well as more specialist and targeted CFT and CBT approaches targeting parental appraisals and behaviors where they have become unhelpful. With such intervention research, it is recommended that outcome measures include more than just child anxiety and focus on parental well-being as well as parental appraisals and behaviors, which will assess the systemic impact of the intervention.

## Supplemental Material

sj-docx-1-cpc-10.1177_10556656261422883 - Supplemental material for The Relationship Between Parent and Child Anxiety: Testing the Intergenerational Model of Anxiety in Children Born with a Cleft Supplemental material, sj-docx-1-cpc-10.1177_10556656261422883 for The Relationship Between Parent and Child Anxiety: Testing the Intergenerational Model of Anxiety in Children Born with a Cleft by Roberta Button, Fiona McFarlane, Alice Farrington and Matthew Hotton in The Cleft Palate Craniofacial Journal

## Data Availability

It is not possible for data to be available to share, as participants did not consent to this.

## References

[1] . CRANE Report. Cleft Registry and Audit NEtwork Database 2023 Annual Report; 2023.

[2] World Health Organization. Addressing the global challenges of craniofacial anomalies. Report of a WHO meeting on International Collaborative Research on Craniofacial Anomalies. Hum Genet Proj WHO Geneva. Published online 2006.

[3] Chadha A Beale V . UK Cleft lip and palate care: A contemporary perspective. Paediatr Child Health. 2023;33(12):382–394. doi:10.1016/j.paed.2023.09.003

[4] Smallridge J Hall AJ Chorbachi R , Parfect V, Persson M, Ireland AJ, Wills AK, Ness AR, Sandy JR. Functional outcomes in the cleft care UK study – part 3: Oral health and audiology. Orthod Craniofac Res. 2015;18(S2):25–35. doi:10.1111/ocr.12110 PMC4950029 26567853

[5] England NHS . National service specification for cleft lip and palate services in England and Wales. Published online 2013.

[6] Branson EK Branson VM McGrath R Rausa VC Kilpatrick N Crowe LM . Psychological and peer difficulties of children with cleft lip and/or palate: A systematic review and meta-analysis. Cleft Palate Craniofac J. 2024;61(2):258–270. doi:10.1177/10556656221125377 36082954

[7] Demir T Karacetin G Baghaki S Aydin Y . Psychiatric assessment of children with nonsyndromic cleft lip and palate. Gen Hosp Psychiatry. 2011;33(6):594–603. doi:10.1016/j.genhosppsych.2011.06.006 21816483

[8] Millar K Bell A Bowman A , Brown D, Lo T-W, Siebert P, Simmons D, Ayoub A. Psychological status as a function of residual scarring and facial asymmetry after surgical repair of cleft lip and palate. Cleft Palate Craniofac J. 2013;50(2):150–157. doi:10.1597/10-222 21846256

[9] Tyler MC Wehby GL Robbins JM Damiano PC . Separation anxiety in children ages 4 through 9 with oral clefts. Cleft Palate Craniofac J. 2013;50(5):520–527. doi:10.1597/11-239 23451755

[10] Brand S Blechschmidt A , Muller A, Sader R, Schwenzer-Zimmerer K, Zeilhofer H-F, Holsboer-Trachsler E. Psychosocial functioning and sleep patterns in children and adolescents with cleft lip and palate (CLP) compared with healthy controls. Cleft Palate Craniofac J. 2009;46(2):124–135. doi:10.1597/07-165.1 19254057

[11] Stock N Feragen KB . Psychological adjustment to cleft lip and/or palate: A narrative review of the literature. Psychol Health. 2016;31(7):777–813. doi:10.1080/08870446.2016.1143944 26800428

[12] Berman SL Weems CF Silverman WK Kurtines WM . Predictors of outcome in exposure-based cognitive and behavioral treatments for phobic and anxiety disorders in children. Behav Ther. 2000;31(4):713–731. doi:10.1016/S0005-7894(00)80040-4

[13] Feragen KB Stock N . When there is more than a cleft: Psychological adjustment when a ceft is associated with an additional condition. Cleft Palate Craniofac J. 2014;51(1):5–14. doi:10.1597/12-328 23621662

[14] Lund C Brooke-Sumner C Baingana F , Baron EC, Breuer E, Chandra P, Haushofer J, Herrman H, Jordans M, *et al*. Social determinants of mental disorders and the sustainable development goals: A systematic review of reviews. Lancet Psychiatry. 2018;5(4):357–369. doi:10.1016/S2215-0366(18)30060-9 29580610

[15] Feragen KB Stock N Kvalem IL . Risk and protective factors at age 16: Psychological adjustment in children with a cleft lip and/or palate. Cleft Palate Craniofac J. 2015;52(5):555–573. doi:10.1597/14-063 25210861

[16] Feragen KB Stock N . Risk and protective factors at age 10: Psychological adjustment in children with a cleft lip and/or palate. Cleft Palate Craniofac J. 2016;53(2):161–179. doi:10.1597/14-062 25794016

[17] Stock N Blaso D Hotton M . Caring for a child with a cleft lip and/or palate: A narrative review. Cleft Palate Craniofac J. Published online September. 2024;9(10556656241280071): 1948–1974. doi:10.1177/10556656241280071 PMC12992653 39246238

[18] Stock N Blaso D White P , Shepherd L, Costa B, Edme K, Aspland R, Hotton M. Longitudinal psychological well-being in caregivers of young children with cleft lip and/or palate. J Pediatr Psychol. Published online May. 2025(9):831–842. doi:10.1093/jpepsy/jsaf029. PMC12451232 40332945

[19] Niinomi K Ueki S Fujita Y , Kitao M, Matsunaka E, Kumagai Y, Ike M. Differences in specific concerns perceived by parents of children with cleft lip and/or palate based on the types of cleft. Int J Paediatr Dent. Published online October. 2021;5:(3):304–313. doi:10.1111/ipd.12886. 34358380

[20] Srivastav S Duggal I Duggal R Tewari N Chaudhari PK Pandey RM . Parental response to the feeding behavior problems in children with cleft lip and palate: A systematic review. Spec Care Dentist. 2021;41(5):559–571. doi:10.1111/scd.12604 33978250

[21] Nelson PA, Kirk SA, Caress A-L, Glenny A-M. Kitao M, Matsunaka E, Kumagai Y, Ike M. Parents’ emotional and social experiences of caring for a child through cleft treatment. Qual Health Res. 2012a;22(3):346–359. 10.1177/1049732311421178 21890716

[22] Lazarus R Folkman S. Stress, Appraisal and Coping. Springer; 1984.

[23] Shuttlewood E Dalton L Cooper M . Developing a measure of appraisal: A psychometric analysis of the parental appraisal of cleft questionnaire. Cleft Palate Craniofac J. 2014;51(2):207–221. doi:10.1597/12-011 23394316

[24] Aktar E . Intergenerational transmission of anxious information processing biases: An updated conceptual model. Clin Child Fam Psychol Rev. Published online February. 2022;26. doi:10.1007/s10567-022-00390-8 PMC8948131 35218453

[25] Creswell C Cooper P Murray L . Intergenerational transmission of anxious information processing biases. In: Hadwin JA Field AP , eds. Information processing biases and anxiety. 1st ed. Wiley; 2010:279–295. doi:10.1002/9780470661468.ch12

[26] Khargekar N Khargekar N Khargekar V Rajan S . Cleft lip and palate—a psychological insight. Sci J Clin Med. 2016;5(4-1):37–40. doi:10.11648/j.sjcm.s.2016050401.17

[27] Pinquart M . Do the parent–child relationship and parenting behaviors differ between families with a child with and without chronic illness? A meta-analysis. J Pediatr Psychol. 2013;38(7):708–721. doi:10.1093/jpepsy/jst020 23660152

[28] Hotton M Shepherd L Stock NM . Cleft lip and palate research in the United Kingdom: Advances in clinical psychological knowledge and future directions. Cleft Palate Craniofac J. Published online January. 2025;28. doi:10.1177/10556656251315659 PMC12996373 39871695

[29] Von Elm E Altman DG Egger M Pocock SJ Gøtzsche PC Vandenbroucke JP . The Strengthening the Reporting of Observational Studies in Epidemiology (STROBE) statement: Guidelines for reporting observational studies. Lancet. 2007;370(9596):1453–1457. doi:10.1016/S0140-6736(07)61602-X 18064739

[30] Chorpita BF Yim L Moffitt C Umemoto LA Francis SE . Assessment of symptoms of DSM-IV anxiety and depression in children: A revised child anxiety and depression scale. Behav Res Ther. 2000;38(8):835–855. doi:10.1016/s0005-7967(99)00130-8 10937431

[31] Donnelly A Fitzgerald A Shevlin M Dooley B . Investigating the psychometric properties of the Revised Child Anxiety and Depression Scale (RCADS) in a non-clinical sample of Irish adolescents. J Ment Health. 2019;28(4):345–356. doi:10.1080/09638237.2018.1437604 29447056

[32] Spitzer RL Kroenke K Williams JBW Löwe B . A brief measure for assessing generalized anxiety disorder: The GAD-7. Arch Intern Med. 2006;166(10):1092. doi:10.1001/archinte.166.10.1092 16717171

[33] Clarke K Cooper P Creswell C . The parental overprotection scale: Associations with child and parental anxiety. J Affect Disord. 2013;151(2):618–624. doi:10.1016/j.jad.2013.07.007 PMC3808745 23916305

[34] Field A . Discovering Statistics Using SPSS. 3rd ed. Sage Publications; 2009.

[35] Cohen J . Statistical Power Analysis for the Behavioural Sciences . 2nd ed. Lawrence Erlbaum Associates; 1988.

[36] Stock N Costa B White P Rumsey N . Risk and protective factors for psychological distress in families following a diagnosis of cleft lip and/or palate. Cleft Palate Craniofac J. 2020;57(1):88–98. doi:10.1177/1055665619862457 31378083

[37] Sischo L Clouston SAP Phillips C Broder HL . Caregiver responses to early cleft palate care: A mixed method approach. Health Psychol. 2016;35(5):474–482. doi:10.1037/hea0000262 PMC4757521 26280177

[38] Stock N Rumsey N . Parenting a child with a cleft: The father’s perspective. Cleft Palate Craniofac J. 2015;52(1):31–43. doi:10.1597/13-035 24295441

[39] Hotton M Cropper J Rundle J Crawford R . The role of the clinical psychologist within a cleft service. Br Dent J. 2023;234(12):887–891. doi:10.1038/s41415-023-5952-0 37349436

[40] Pinckston M Dalton L Farrar S Hotton MT . The psychosocial adjustment of children born with a cleft lip and/or palate: Cross-sectional and longitudinal analyses. Cleft Palate Craniofac J. 2020;57(11):1280–1290. doi:10.1177/1055665620921669 32383406

[41] Kazak AE Scialla M Deatrick JA Barakat LP . Pediatric psychosocial preventative health model: Achieving equitable psychosocial care for children and families. Fam Syst Health. 2024;42(1):76–89. doi:10.1037/fsh0000856 38010765

[42] Gilbert P . The origins and nature of compassion focused therapy. Br J Clin Psychol. 2014;53(1):6–41. doi:10.1111/bjc.12043 24588760

[43] Sirois FM Bögels S Emerson LM . Self-compassion improves parental well-being in response to challenging parenting events. J Psychol. 2019;153(3):327–341. doi:10.1080/00223980.2018.1523123 30376651

[44] Creswell C Violato M Fairbanks H , White E, Parkinson M, Abitabile G, Leidi A, Cooper PJ. Clinical outcomes and cost-effectiveness of brief guided parent-delivered cognitive behavioural therapy and solution-focused brief therapy for treatment of childhood anxiety disorders: A randomised controlled trial. Lancet Psychiatry. 2017;4(7):529–539. doi:10.1016/S2215-0366(17)30149-9 PMC5483485 28527657

[45] Norman A Persson M Stock N , Rumsey N, Sandy J, Waylen A, Edwards Z, Hammond V, Partridge L, Ness A. The effectiveness of psychosocial intervention for individuals with cleft lip and/or palate. Cleft Palate Craniofac J. 2015;52(3):301–310. doi:10.1597/13-276 24878344

[46] Manley S Francis S . The role of parental anxiety sensitivity and beliefs about child anxiety in the relationship between parent and child anxiety. J Psychopathol Behav Assess. 2022;44(1):125–138. doi:10.1007/s10862-021-09937-5

[47] Serafimova T Loades M Gaunt D Crawley E . Who should we ask about mental health symptoms in adolescents with CFS/ME? Parent-child agreement on the revised children’s anxiety and depression scale. Clin Child Psychol Psychiatry. 2021;26(2):367–380. doi:10.1177/1359104521994880 PMC8172254 33586480

[48] Crerand CE Conrad AL Bellucci CC , Albert M, Heppner CE, Sheikh F, Woodard S, Udaipuria S, Kapp-Simon KA. Psychosocial outcomes in children with cleft lip and/or palate: Associations of demographic, cleft morphologic, and treatment-related variables. Cleft Palate Craniofac J. 2024;61(11):1784–1802. doi:10.1177/10556656231181581 37350106

