# Supplementary material for: The Relationship Between Parent and Child Anxiety: Testing the Intergenerational Model of Anxiety in Children Born with a Cleft
Source: Cleft Palate Craniofac J. 2026 Mar 23;63(11):3707–16. doi: 10.1177/10556656261422883 (PMC13646421; doi:10.1177/10556656261422883)
Supplement: Supplementary file 1 [file sj-docx-1-cpc-10.1177_10556656261422883.docx]

**Supplemental Table 1. Skewness and Kurtosis of significantly non-normally distributed data**

|  | **Skewness** | **Kurtosis** |
| --- | --- | --- |
| **Child age** | 0.18 | -1.136 |
| **RCADS-P** | 1 | 0.79 |
| **GAD-7** | 0.67 | -.43 |
| **OP** | 0.53 | -0.95 |
| **PACQ subscale 2** | 0.37 | -0.39 |
| **PACQ subscale 4** | -1.6 | 2.28 |
| **PACQ subscale 5** | -0.5 | -0.6 |
